# Newspaper coverage before and after the HPV vaccination crisis began in Japan: a text mining analysis

**DOI:** 10.1186/s12889-019-7097-2

**Published:** 2019-06-17

**Authors:** Tsuyoshi Okuhara, Hirono Ishikawa, Masafumi Okada, Mio Kato, Takahiro Kiuchi

**Affiliations:** Department of Health Communication, School of Public Health, The University of Tokyo, 7-3-1 Hongo, Bunkyo-ku, Tokyo, 113-8655 Japan

**Keywords:** HPV vaccine, Human papillomavirus vaccine, Anti-vaccination movement, Cervical cancer, Newspaper, Content analysis, Text mining

## Abstract

**Background:**

The human papillomavirus (HPV) vaccination coverage rate has fallen sharply in Japan since 2013, when newspapers began covering negative campaigns against the vaccination. We examined and compared contents from newspaper articles before and after the start of this HPV vaccination crisis.

**Methods:**

We collected articles published between January 2005 and September 2017 in the four daily national Japanese newspapers with the highest domestic circulation. We then conducted text mining analysis to chronologically examine content distribution.

**Results:**

From among the 1178 articles analyzed, 12 types of contents were identified. Contents related to cervical cancer prevention, such as on the risk of developing cervical cancer, causes of cervical cancer, and the effects of vaccination, were frequently conveyed until 2012. However, after March 2013, they were replaced with anti-vaccination contents, such as on adverse effects to vaccines, alleged victims, and related lawsuits. Meanwhile pro-vaccination contents, such as safety statements from the World Health Organization, scarcely received coverage.

**Conclusions:**

Newspaper contents changed profoundly before and after the start of the vaccination crisis. Those newspaper reports potentially had impact on readers’ beliefs and actions. Journalists should strive for impartial coverage so readers can make more-informed decisions. Health professionals should be expected to work with journalists to help improve impartiality in newspaper coverage. The Ministry of Health, Labour and Welfare should discus benefits and risks of the HPV vaccination based on the scientific evidences, and consider to resume the proactive recommendation of HPV vaccination. Well-organized advocacy among medical societies, scientists and health professionals will also be needed to influence the government.

## Background

The media play a key role in setting the agenda for various issues, and news stories frequently take on a particular perspective [1]. In this sense, the media also affect perceptions of health issues in the eyes of the public and policymakers [2].

Media coverage of vaccination issues can affect parents’ decision making on having their children vaccinated, and can consequently affect vaccine uptake. Media campaigns that positively promote immunization have been shown to increase public knowledge of the dangers of vaccine-preventable diseases, and to improve vaccine uptake rates [3, 4]. Conversely, still fresh in many people’s memories is the adverse publicity concerning the safety of the measles, mumps, and rubella (MMR) vaccine in the UK, which started with the 1998 publication of a fraudulent research paper. This impelled large numbers of parents to refuse immunization for their children [5, 6]. Similarly, the human papillomavirus (HPV) vaccination rate has now stagnated in Japan, and media coverage of the vaccination may have played a substantial role in this outcome.

Cervical cancer, which is commonly caused by chronic infection with an oncogenic strain of HPV, is the third most commonly diagnosed cancer and the fourth leading cause of cancer deaths among women worldwide [7, 8]. Approximately 10,000 people are diagnosed with, and about 3000 people die of, cervical cancer annually in Japan [9]. Cervical cancer incidence and mortality have increased in recent years among women in their 20s and 30s [9]. HPV vaccination is recommended by the World Health Organization (WHO) [10] and has been made available in most industrialized countries. The Ministry of Health, Labour and Welfare of Japan approved the manufacture and sale of the vaccines Cervarix in 2009 and Gardasil in 2011. Additionally, the Ministry of Health, Labour and Welfare and municipalities began subsidizing costs of HPV vaccination in 2010 (see Appendix 1 for major events surrounding HPV vaccination in Japan).

The HPV vaccination rate for girls aged 12–16 years was as high as about 70% in 2011 and 2012 in Japan [11, 12]. However, in March 2013, *The Asahi Shimbun*, considered one of the most authoritative newspapers in Japan, reported on a girl who had allegedly suffered from severe adverse effects attributed to the HPV vaccine. Newspapers, television, and other media followed suit, and continuously reported on adverse events of HPV vaccination, including movement disorders and memory disturbances. Although HPV vaccines became a routine prophylactic vaccine under Japan’s Preventive Vaccination Law in April 2013, the Japanese government decided to suspended its proactive recommendation of HPV vaccination in June 2013, in consideration of public concerns about those adverse events. As a result, the HPV vaccination rate fell sharply, to only a few percent by 2014 [13, 14].

In this way, the HPV vaccination crisis in Japan began with sensational newspaper articles implying there could be severe adverse reactions to HPV vaccines [15]. Newspaper readership remains strong in Japan, as the Japan Newspaper Publishers and Editors Association indicated 78% of Japanese people read a newspaper occasionally and 64% read one every morning; among which roughly half of people aged 15–29 also occasionally read newspapers as of an 2015 report [16]. In a Japanese survey in 2016 that asked participants their perceived proportion of credible information in newspapers, television, internet, and magazines using a five point scale, the perceived credibility of media outlets among teens to people in their 60s was highest for newspapers, followed by television, then the internet [17].

Studies show limited knowledge about HPV infection, HPV vaccines, and cervical cancer, and concerns regarding the safety of HPV vaccines are among factors associated with parents’ and daughters’ hesitant attitudes toward HPV vaccines inside and outside Japan [18–27]. Newspapers, depending on their contents, are capable of increasing HPV-related knowledge, or of instilling fear of adverse reactions to HPV vaccination. Newspaper coverage therefore evidently influenced people’s perceptions of HPV vaccination in Japan.

Previous content analysis of newspaper articles about HPV vaccination in Western countries reported that newspaper content generally showed a positive tone [28–31], despite lacking detailed health-related information about HPV [4, 31–33]. One study revealed that Japanese newspaper articles about HPV vaccination after March 2013 were more likely to have included adverse-reaction-related keywords and more frequently had a negative tone compared with those published before March 2013 [15]. However, as far as we know, nothing is known on the precise contents from Japanese newspapers with regard to HPV vaccines. We used a text mining method to examine contents of Japanese newspaper articles about HPV vaccination before and after the crisis that began with the first article in March 2013. We also present a discussion of potential impact that those newspaper reports may have had on readers, based on factors previous studies have found associated with hesitancy toward HPV vaccines.

## Methods

### Data collection

We collected the paper version of articles from the four daily national Japanese newspapers with the highest circulation in Japan, totaling about 20 million: *The Asahi Shimbun*, *The Yomiuri Shimbun*, *The Mainichi Shimbun*, and *The Nikkei*. Article data were obtained from the respective archive services for each newspaper: Kikuzo Visual (http://database.asahi.com/); Yomidasu Rekishikan (https://database.yomiuri.co.jp/); Maisaku database (http://mainichi.jp/contents/edu/maisaku); and Nikkei Telecom 21 (http://t21.nikkei.co.jp). Although the English language has words including vaccine, vaccination, and immunization, the Japanese language only contains *wakuchin* (vaccine) and *yoboseshu* (vaccination or immunization). Additionally, Japanese refer to HPV vaccine as *shikyu keigan wakuchin* (cervical cancer vaccine). Therefore, our search was performed using the Japanese keywords “*shikyu keigan wakuchin*” OR “*shikyu keigan yoboseshu*” OR “*shikyu keigan yobo wakuchin*” (*yobo* means prevention). We input the commonly used Japanese characters (*kanji* and *katakana*) that meant those keywords into the databases. We searched full text (headings and texts) of articles including those keywords. The dates of HPV vaccine introduction were 2006 in Australia and Canada; 2007 in France, Germany, Italy, and South Korea etc. We considered that the Japanese newspapers may have reported the events regarding HPV vaccination in those foreign countries. Therefore, we conducted our search from January 2005 to September 2017.

### Coding procedure

Initially, the first author thoroughly read textual data of all articles identified to grasp the ideas expressed. We then analyzed the data by using a text mining method using KH Coder Version 2.00f (Higuchi, Ritsumeikan University, Kyoto, Japan) [34, 35] software for quantitative content analysis. The software, which supports Japanese text, uses the ChaSen Morpho-logical Analyzer and R statistical software environment. KH Coder has been successfully used in public health studies both in and outside of Japan [36, 37]. The method using KH Coder as follows was successfully applied in previous studies [38, 39].

KH Coder read the data and showed a total of 165,517 terms analyzed, 9288 unique terms analyzed, and 8091 paragraphs analyzed. For clarity of analysis, we excluded common general terms (e.g., “this,” “it,” or “think”) before performing analysis. First, we conducted hierarchical cluster analysis (Ward’s method) to examine the appearance pattern of terms [40, 41]. The calculation unit was one paragraph. Analysis results were presented using a dendrogram, within which lines were drawn to show clusters of terms close in their appearance pattern. This analysis helped in exploring how the terms were used in the materials. We then conducted correspondence analysis to examine the characteristic terms in articles before the March 2013 article and after it. Analytical results were presented in a two-dimensional graphical form, within which terms were placed depending on the strength of relationship with articles before and after the March 2013 article. This analysis helped to explore terms that characterized contents of these articles.

Additionally, we extracted the top 100 terms in order of higher probability of appearance in articles before and after the March 2013 article. We then conducted network analysis of examined co-occurrence of those terms [40, 41]. Analytical results were presented in the figure of a network, within which terms with a great degree of co-occurrence were linked to each other. The degree of co-occurrence was determined using the Jaccard similarity coefficient. This analysis helped in exploring the appearance pattern of terms, as well as the contents the linked terms represented. In these ways, we narrowed the characteristic terms in articles before and after the March 2013 article, and the contents that those co-occurring terms represented.

We created coding rules representing specific contents by combining frequently appearing co-occurring terms in articles before and after the March 2013 article. Coding rules are combinations of terms and logical operators, such as “and,” “or,” “and not,” and “or not.” For example, a coding rule to extract paragraphs referring to “benefit and risk” of medical practice could be: “(benefit or advantage or merit) and (risk or disadvantage or demerit).” We sought to create as many codes as possible to exhaustively examine frequently appearing contents. We conducted trial analyses, reviewed code-fitted paragraphs, and revised the coding rules by adding or deleting terms and rewriting logical operators for greater accuracy. The first author repeated these procedures (i.e., creating and revising the coding rules) twice, with a 3-week interval between analyses to ensure consistency. These procedures allowed us to examine closely all relevant terms (e.g., synonyms) and irrelevant terms, and comprehensively select relevant paragraphs and avoid irrelevant ones. Finally, we defined 12 codes (Table 1), which were classified into three categories depending on their content: prevention of cervical cancer, risk of HPV vaccination, and attitudes of expert organizations. After the research was completed, we translated all terms into English for the purpose of this report.

**Table 1:** Definitions of codes

Categories	Codes	Contents	Examples of terms used in coding rules^a^
Prevention of cervical cancer	Risk of developing cervical cancer	References cervical cancer incidence and mortality rate.	incidence, morbidity, mortality, develop, death, year
	Cause of cervical cancer	Viral infection is the cause of cervical cancer.	cause, virus, infection
	Effect of HPV vaccination	Vaccination can prevent HPV infection and protect women from cervical cancer.	prevent, infection, effect
	Subsidy for HPV vaccination cost	The Ministry of Health, Labour and Welfare and municipalities support costs of HPV vaccination.	subsidy, public expense, free of charge
	Cancer screening	Obtaining cancer screening is recommended.	cancer screening
Risk of HPV vaccination	Adverse effects	References adverse effects of HPV vaccination.	side effect, adverse effects, adverse event, intense pain, disorder
	Suspension of proactive recommendation of vaccination	Ministry of Health, Labour and Welfare temporarily suspends proactive recommendation of HPV vaccination.	recommendation, suspension, cancellation
	Vaccine-induced damage	References vaccine-induced damage and victims.	drug-induced damage, victim
	Lawsuit	Girls who suffer from adverse effects, and their families, sued the Ministry of Health, Labour and Welfare and pharmaceutical companies.	suit, accuser, court, indemnity
Attitudes of expert organizations	Safety statement	Statement from expert committees, such as the World Health Organization (WHO), on safety of HPV vaccines	WHO, academic organization, statements
	Mind–body reaction	The expert opinion review meeting in the Ministry of Health, Labour and Welfare stated that the cause of symptoms after the HPV vaccination is a “mind–body reaction.”	mind–body reaction, psychosomatic
	Relief for sufferers from adverse effects	The Ministry of Health, Labour and Welfare pays medical expenses to those who suffer from adverse effects after HPV vaccination.	relief, compensation, support, medical expense
	Survey on adverse effects	References a survey conducted in Nagoya comparing vaccinated persons and non-vaccinated individuals on symptoms of adverse effects in approximately 30,000 subjects.	Nagoya, survey

^a^The authors translated terms from Japanese to English for the purpose of this report

### Analysis

Descriptive statistics, including frequencies and percentages, were used to calculate and summarize the data. The distribution of paragraphs that fit into each code (i.e., appearance ratios of code-fitted paragraphs with the total number of code-fitted paragraphs as the numerator and total number of paragraphs analyzed as the denominator) was calculated every 3 months, as a time unit.

## Results

**Fig. 1:**
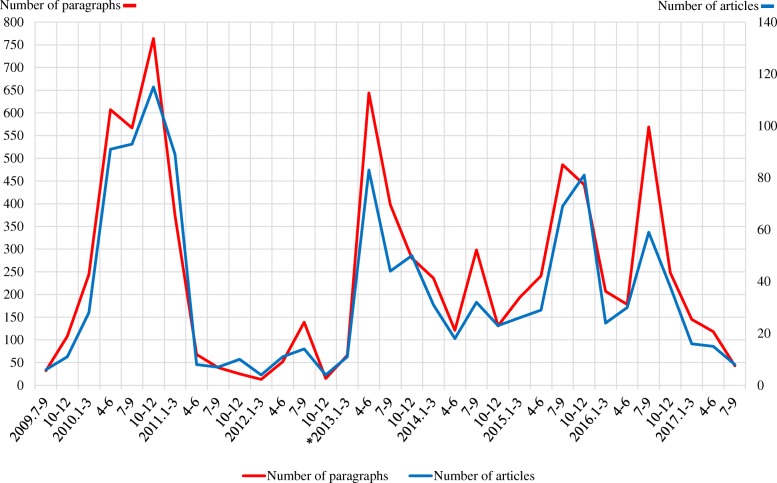
Numbers of HPV-vaccination-related articles in the major Japanese newspapers from July 2009 to September 2017. *Severe adverse effects from the HPV vaccine were first reported in a newspaper in March 2013

We identified 1178 articles that concerned HPV vaccination. Of these articles, 413 were in *The Asahi Shimbun*, 360 in *The Yomiuri Shimbun*, 298 in *The Mainichi Shimbun*, and 107 in *The Nikkei*. Figure 1 shows the number of paragraphs and articles from July 2009 to September 2017 (see Appendix 2 for data). No articles were identified between January 2005 and July 2009. Among the examined articles, HPV vaccination was first reported in August 2009. Figure 1 shows four peaks of number of articles and paragraphs: around November 2010 at the start of public subsidies for HPV vaccination; in April–June 2013 with continuous media reports about severe adverse effects of HPV vaccination, start of free HPV vaccination based on the public expenditure system, and suspension of the government’s proactive recommendation of HPV vaccination; around September 2015 when the government decided to pay medical expenses to those who suffered from adverse effects; and around July 2016 at time individuals who suffered from adverse effects filed lawsuits.

**Fig. 2:**
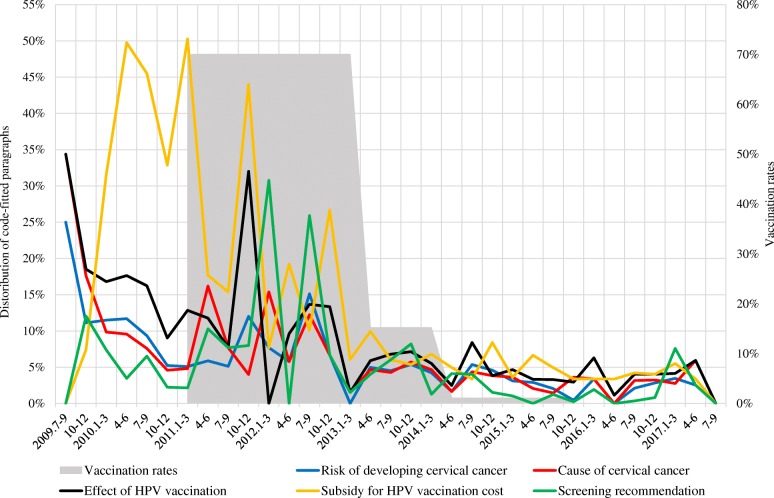
Distribution of code-fitted paragraphs related to prevention of cervical cancer

**Fig. 3:**
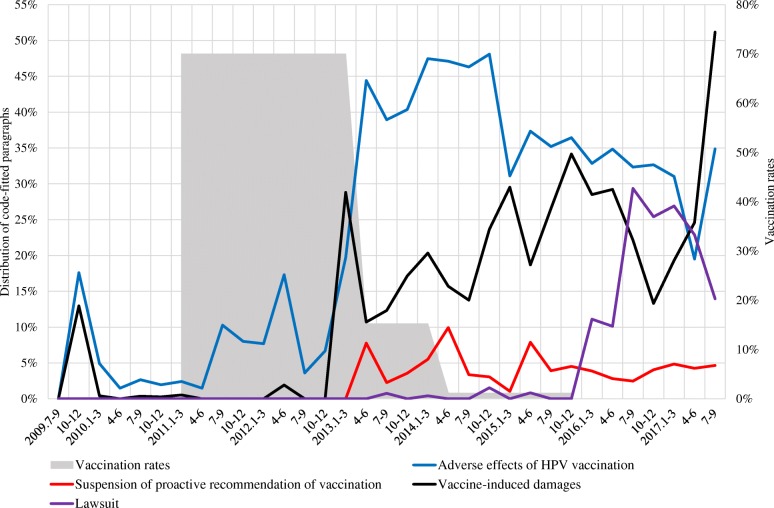
Distribution of code-fitted paragraphs related to risk of HPV vaccination

**Fig. 4:**
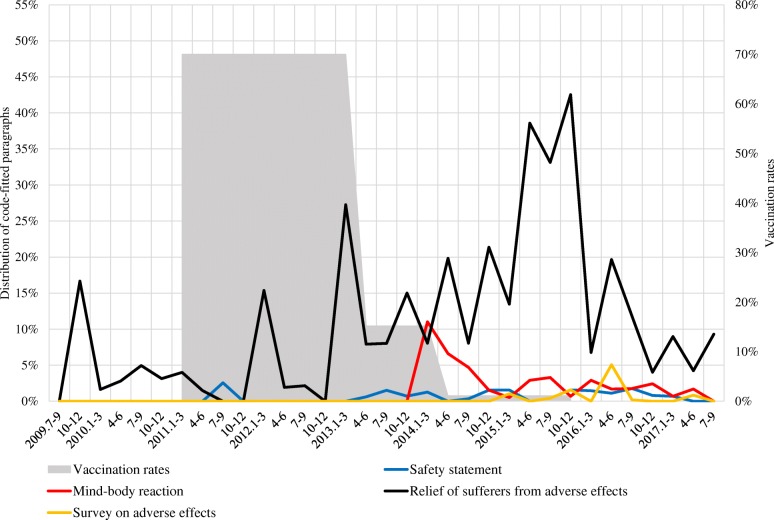
Distribution of code-fitted paragraphs related to attitudes of expert organizations

Figs. 2, 3, 4 show the distribution of code-fitted paragraphs over time periods, and the rates of three-dose completion of HPV vaccination for girls aged 12–16 years in 2011–2015 (approximate rates based on several surveys, as there was no follow-up survey encompassing the entire period) [11, 12, 14, 42, 43].

Codes about prevention of cervical cancer are in Fig. 2 (see Appendix 3 for data). Figure 2 shows that paragraphs including terms related to cervical cancer prevention (i.e., risk of developing cervical cancer [blue line], cause of cervical cancer [red line], screening recommendation [green line], effect of HPV vaccine [black line], and subsidy for HPV vaccination cost [yellow line]) occupied relatively large proportions in the articles before the March 2013 article compared with those after. In particular, paragraphs referring to subsidies for the HPV vaccination cost frequently appeared before the March 2013 article.

Codes about risk of HPV vaccination are in Fig. 3 (see Appendix 3 for data). Figure 3 shows the proportion of paragraphs including terms related to risk of HPV vaccination (blue line) and terms such as “vaccine-induced damage” and “victims” (black line) increased after March 2013. Additionally, paragraphs related to lawsuits (purple line) increased from 2016.

Codes about attitudes of expert organizations are in Fig. 4 (see Appendix 3 for data). Figure 4 shows the distribution of paragraphs including terms related to expert organizations such as the Ministry of Health, Labour and Welfare, municipalities, academic societies, and the WHO. Paragraphs referring to the government’s view that the cause of symptoms after HPV vaccination was a psychosomatic “mind–body reaction” (red line) increased only in the first half of 2014, then decreased from that time. The proportion of paragraphs referring to safety statements from the WHO and academic societies (blue line), and the proportion of paragraphs referring to survey on adverse effects were consistently very small. Paragraphs referring to government comments on relief of sufferers from adverse effects to HPV vaccines frequently appeared in 2015.

## Discussion

This study quantitatively examined contents of Japanese newspaper articles about HPV vaccination over the period covering January 2005 to September 2017. The number of paragraphs and articles tended to increase at the time major events were reported from 2010 to 2016. However, number of articles and paragraphs decreased in 2017. This should be noted because it may reflect journalists’ decreasing interest and may indicate the general public will in the near future forget HPV vaccination as a past scandal, despite this is the first case of strong vaccine hesitancy in Japan driven by safety concerns.

Fig. 2 shows that the paragraphs occupied relatively large proportions in the articles before the March 2013 article. These paragraphs were considered to have reported HPV vaccines in a positive tone as a new option for prevention of cervical cancer. This trend of a positive tone in news articles during the period immediately pre- and post-approval of HPV vaccines was consistent with previous studies that analyzed news articles about these vaccines in Western countries [28–31]. Studies outside Japan found positive media campaigns to promote immunization increased public knowledge and improved vaccine uptake rates [3, 4]. In Japan as well, the positive contents about HPV vaccination may have contributed to the high vaccination rate of 70–80% until 2012 [11]. However, after March 2013, paragraphs including terms related to cervical cancer prevention decreased sharply. This decrease may have resulted in limited knowledge about HPV infection and cervical cancer, and these limitations may have become barriers to HPV vaccination after March 2013 [18–27].

As Fig. 3 shows, the predominance of paragraphs referring to risk of HPV vaccination after the March 2013 article is in striking contrasts with the frequent appearance of paragraphs referring to cervical cancer prevention until 2012. Dominant frames of media coverage often define what issues audiences view as important [1]. Studies have reported that Japanese mothers, who are the main decision-makers in relation to the HPV vaccination for their daughters [18, 42], underestimate the morbidity and mortality associated with cervical cancer [18], and overestimate the probability of severe adverse effects 10–1000-fold [42]. Newspaper articles focusing on the adverse effects of HPV vaccines after March 2013 may have helped define the audience’s views and contributed to formation of false perceptions regarding the vaccines. Studies show that concern about adverse effects is a strong barrier to HPV vaccination [18–27, 42]. Articles focused on adverse effects may have encouraged readers to forgo vaccination.

In this situation in which the causal relationship between the HPV vaccination and symptoms after vaccination was unclear, the newspaper articles that frequently used terms such as “vaccine-induced damage” and “victims” should be subject to criticism. That is because descriptive language such as “the symptoms may be adverse side effects of HPV vaccines” is neutral, though “damage” and “victims” strongly suggest a causal relationship between vaccination and subsequent symptoms. Articles using such terms likely stoked readers’ fears of adverse effects. Paragraphs referring to suspension of proactive recommendation of HPV vaccination (red line) likely induced doubts about safety among readers. Paragraphs including terms related to lawsuits, which increased from 2016 (purple line), probably strengthened anti-HPV-vaccination sentiment.

Figure 4 shows that only a little of attitudes of expert organizations regarding safety of vaccines has been reported. The WHO’s Global Advisory Committee of Vaccine Safety (GACVS) criticized the shift in Japan’s HPV vaccine policy in March 2014, stating that “policy decisions based on weak evidence, leading to lack of use of safe and effective vaccines, can result in real harm.” [44]. The GACVS also reaffirmed this opinion in December 2015, stating that “young women are being left vulnerable to HPV-related cancers that otherwise could be prevented” [45]. However, among the examined newspapers, these opinions from the WHO were scarcely conveyed to Japanese public. This may have contributed to readers’ avoidance of the vaccines, considering that a lack of health professionals’ recommendations is associated with hesitancy toward the vaccines [18–27].

The city of Nagoya conducted a survey in September 2015 to compare HPV-vaccinated and non-vaccinated individuals regarding the appearance of 24 alleged symptoms of adverse reactions to vaccination, encompassing approximately 30,000 individuals. Nagoya announced in the initial report of the results in December 2015 that there was no significant increase of symptoms among HPV-vaccinated individuals compared with those not vaccinated. However, only a very small (yellow line) proportion of paragraphs referred to these survey results around December 2015. Victim support organizations and anti-HPV-vaccine activists directed harsh online and offline criticism at Nagoya, alleging the study had flawed methodology and the results could not be trusted. Nagoya announced the final report from the study in June 2016. However, that report only released data and had no statistical comparisons between groups. Moreover, it did not refer to the causal relationship between HPV vaccination and symptoms. This weak attitude by the city was considered to have been for the sake of avoiding protest by anti-HPV vaccine activists. Accordingly, such protesting may have silenced scientific findings. Noteworthy here is that the proportion of paragraphs referring to the final report in June 2016 exceeded the proportion of paragraphs referring to the initial report in December 2015. The examined newspapers reported the weak final report more than the initial report implying no causal relationship between vaccination and symptoms.

Paragraphs referring to government comments on relief of sufferers from adverse effects to HPV vaccines frequently appeared in 2015. Readers may have interpreted those reports as the government acknowledging the causal relationship between HPV vaccines and symptoms after vaccination. This was quite likely to have strengthened their doubts about HPV vaccines’ safety and their vaccine avoidance.

Thus, the results of the present study indicate the examined newspapers frequently conveyed negative contents about HPV vaccines, such as adverse effects, vaccine-induced damages, and lawsuit after March 2013. Conversely, over the same period, they rarely conveyed positive contents about the vaccines, such as on cervical cancer prevention, positive effects of the vaccines, safety statements by the WHO, and the Nagoya study results. Considering the factors associated with hesitancy toward the HPV vaccines, such as in concern about the vaccines’ safety, as well as limited HPV-related knowledge [18–27], this biased newspaper coverage may have contributed to the sharp decline in the HPV vaccination rate from 2013 (gray area in Figs. 2, 3, 4). Although Japanese newspapers have criticized the safety of HPV vaccines and the government’s HPV vaccination policy, they may in turn be criticized in future for putting young women at risk of otherwise preventable HPV-related cancers.

Ways of avoiding bias in newspaper coverage were out of the scope of this study, though we can offer some suggestions. One study interviewed journalists to explore how they select and shape news on health issues, and offered recommendations for public health professionals to work more effectively with the media; these included, being readily accessible for journalists, cultivating specialist medical reporters, providing reliable and useful information resources [46]. These recommendations may also be applicable to reducing bias in Japanese newspaper coverage of HPV vaccination. For example, Japanese health professionals could be expected to more actively hold press conferences and study meetings about HPV vaccination with journalists to build relationships with them, cultivate specialists, and provide resources. Additionally, increasing the number of health professionals who have direct and unfiltered input into issues via outlets such as blogging and Twitter may help promote and advocate for HPV vaccination using the strengths of online social distribution and increasing accessibility for journalists online. Health professionals’ proactive grassroots communication with journalists, in addition to authoritative public relations through academic societies, may be needed to directly appeal to journalists’ consciences, attract their attention, and improve on their negatively biased reporting on HPV vaccination.

In considering the importance of policy making, it may be helpful to refer a passed stagnation of influenza vaccination for school children in Japan. Under the Preventive Vaccination Law, more than 10 million schoolchildren received influenza vaccine annually from 1976 to 1987, with a peak of 16.5 million vaccines [47]. However, seasonal epidemics continued to occur, which resulted in an anti-vaccination campaign claiming that influenza vaccine was ineffective. Influenza vaccine coverage among schoolchildren declined sharply from about 80% at its peak to 18% in 1992. In 1994, the Preventive Vaccination Law was amended to exclude influenza from the list of target diseases. Influenza vaccine coverage had remained to be quite low until the Preventive Vaccination Law was amended in 2001 to again include influenza. No to make the same mistake as that of influenza vaccination, the Ministry of Health, Labour and Welfare should resume the proactive recommendation of HPV vaccination as soon as possible.

Medical societies, scientists, and health professionals have tried influence the government to a certain degree. For example, the Japan Pediatric Society submitted a written request for resuming the proactive recommendation of HPV vaccination to the Ministry of Health, Labor and Welfare. Additionally, 15 Japanese academic organizations such as the Japan Pediatric Society, Japan Society of Obstetrics and Gynecology, and Japanese Association for Infectious Diseases jointly called for prompt resumption of active vaccine recommendation. Some scientists and health professionals positively have presented their opinions for resuming the proactive recommendation of HPV vaccination on the internet and social media. Despite those effort of experts, the Japanese government has yet to resume endorsement of HPV vaccination as of November 2018. Beyond a mere cooperation and discussion among medical societies, scientists, and health professionals, well-organised advocacy to influence the government for resuming the proactive recommendation of HPV vaccination — emphasizing safety more strongly and responding robustly to misinformation — will be needed such as an example occurred in Ireland [48].

The present study has some limitations. The present study quantitatively examined distribution of code-fitted paragraphs, though did not qualitatively examine details of contents and the context of those paragraphs. Because the present study is a text mining analysis using a software, we could not identify whether each articles were one-sided or two-sided arguments. Creation of coding rules may have reflected biases on the part of the authors. The analysis of the present study did not include the online editions of newspaper articles. Additionally, the present study did not take into account the role of social media and particular lobby groups, which may have played an important role in propagating negative messages and the loss of confidence in the HPV vaccine in Japan [49]. The influence of newspaper articles on readers’ perceptions is unknown, and such examination may be beneficial in future studies. Despite the limitations, the present study has significant implications, as detailed above.

## Conclusions

The present study showed that the Japanese newspaper contents about HPV vaccines strikingly changed around March 2013. Contents related to cervical cancer prevention were frequently conveyed until 2012. However, they suddenly decreased after March 2013. Negative contents, such those on as adverse effects and vaccine-induced damage, became the dominant voice, while positive contents, such as safety statements by the WHO, were rarely conveyed after the March 2013 article that began the trend.

Future research should continue to monitor newspaper coverage of HPV vaccines to assess this coverage for bias. Newspaper journalists should be expected to strive for impartial coverage so readers can make more-informed decisions regarding HPV vaccination. Health professionals should be expected to make efforts, such as more actively holding press conferences and study meetings with journalists, to help improve impartiality in newspaper coverage. Well-organized advocacy among medical societies, scientists and health professionals will also be needed to influence the Ministry of Health, Labour and Welfare for resuming the proactive recommendation of HPV vaccination.

## Data Availability

The datasets used and/or analysed during the current study are available from the corresponding author on reasonable request.

## Appendix 1

**Table 2:** Major events regarding HPV vaccination in Japan

2009	October	Ministry of Health, Labour and Welfare approves manufacture and sale of Cervarix
2010	November	Ministry of Health, Labour and Welfare and municipalities begin subsidizing costs of HPV vaccination
2013	March	*The Asahi Shimbun* reports a case of severe adverse effects to the HPV vaccine, and media continue coverage
		Press conference by organization of alleged victims of HPV vaccines held for the first time
	April	Free HPV vaccination based on public expenditure system based on the immunization law starts
	June	Ministry of Health, Labour and Welfare suspends proactive recommendation of HPV vaccination
2014	January	Expert opinion review meeting in Ministry of Health, Labour and Welfare states the cause of symptoms after HPV vaccination is a psychosomatic “mind–body reaction”
2015	September	Ministry of Health, Labour and Welfare decides to pay medical expenses to those who suffer from side effects after HPV vaccination
2016	July	Girls who suffer from side effects, and their families, sue Ministry of Health, Labour and Welfare and two pharmaceutical companies

## Appendix 2

**Table 3:** Numbers of articles and paragraphs over the time periods

Time period	Number of paragraphs	Number of articles
2009.7–9	32	6
10–12	108	11
2010.1–3	244	28
4–6	607	91
7–9	567	93
10–12	764	115
2011.1–3	374	89
4–6	68	8
7–9	39	7
10–12	25	10
2012.1–3	13	4
4–6	52	11
7–9	139	14
10–12	15	4
2013.1–3	66	11
4–6	644	83
7–9	398	44
10–12	280	50
2014.1–3	236	31
4–6	121	18
7–9	298	32
10–12	131	23
2015.1–3	193	26
4–6	241	29
7–9	486	69
10–12	442	81
2016.1–3	207	24
4–6	178	30
7–9	569	59
10–12	248	38
2017.1–3	145	16
4–6	118	15
7–9	43	8
Total	8091	1178

## Appendix 3

**Table 4:** Distribution of code-fitted paragraphs over time periods

	Risk of developing cervical cancer *n* (%)	Cause of cervical cancer *n* (%)	Effect of HPV vaccination *n* (%)	Subsidy for HPV vaccination cost *n* (%)	Screening recommendation *n* (%)	Adverse effects of HPV vaccination *n* (%)	Suspension of proactive recommendation of vaccination *n* (%)	Vaccine–induced damages *n* (%)	Lawsuit *n* (%)	Safety statement *n* (%)	Mind–body reaction *n* (%)	Relief for sufferers from side effects *n* (%)	Survey on adverse effects *n* (%)	Total paragraphs
2009.7–9	8 (25.0)	11 (34.4)	11 (34.4)	0 (0.0)	0 (0.0)	0 (0.0)	0 (0.0)	0 (0.0)	0 (0.0)	0 (0.0)	0 (0.0)	0 (0.0)	0 (0.0)	32
2009.10–12	12 (11.1)	19 (17.6)	20 (18.5)	8 (7.4)	13 (12.0)	19 (17.6)	0 (0.0)	14 (13.0)	0 (0.0)	0 (0.0)	0 (0.0)	18 (16.7)	0 (0.0)	108
2010.1–3	28 (11.5)	24 (9.8)	41 (16.8)	77 (31.6)	18 (7.4)	12 (4.9)	0 (0.0)	1 (0.4)	0 (0.0)	0 (0.0)	0 (0.0)	4 (1.6)	0 (0.0)	244
2010.4–6	71 (11.7)	58 (9.6)	107 (17.6)	302 (49.8)	21 (3.5)	9 (1.5)	0 (0.0)	0 (0.0)	0 (0.0)	0 (0.0)	0 (0.0)	17 (2.8)	0 (0.0)	607
2010.7–9	53 (9.4)	43 (7.6)	92 (16.2)	258 (45.5)	37 (6.5)	15 (2.7)	0 (0.0)	2 (0.4)	0 (0.0)	0 (0.0)	0 (0.0)	28 (4.9)	0 (0.0)	567
2010.10–12	40 (5.2)	35 (4.6)	69 (9.0)	251 (32.9)	17 (2.2)	15 (2.0)	0 (0.0)	2 (0.3)	0 (0.0)	0 (0.0)	0 (0.0)	24 (3.1)	0 (0.0)	764
2011.1–3	19 (5.1)	18 (4.8)	48 (12.8)	188 (50.3)	8 (2.1)	9 (2.4)	0 (0.0)	2 (0.5)	0 (0.0)	0 (0.0)	0 (0.0)	15 (4.0)	0 (0.0)	374
2011.4–6	4 (5.9)	11 (16.2)	8 (11.8)	12 (17.7)	7 (10.3)	1 (1.5)	0 (0.0)	0 (0.0)	0 (0.0)	0 (0.0)	0 (0.0)	1 (1.5)	0 (0.0)	68
2011.7–9	2 (5.1)	3 (7.7)	3 (7.7)	6 (15.4)	3 (7.7)	4 (10.3)	0 (0.0)	0 (0.0)	0 (0.0)	1 (2.6)	0 (0.0)	0 (0.0)	0 (0.0)	39
2011.10–12	3 (12.0)	1 (4.0)	8 (32.0)	11 (44.0)	2 (8.0)	2 (8.0)	0 (0.0)	0 (0.0)	0 (0.0)	0 (0.0)	0 (0.0)	0 (0.0)	0 (0.0)	25
2012.1–3	1 (7.7)	2 (15.4)	0 (0.0)	1 (7.7)	4 (30.8)	1 (7.7)	0 (0.0)	0 (0.0)	0 (0.0)	0 (0.0)	0 (0.0)	2 (15.4)	0 (0.0)	13
2012.4–6	3 (5.8)	3 (5.8)	5 (9.6)	10 (19.2)	0 (0.0)	9 (17.3)	0 (0.0)	1 (1.9)	0 (0.0)	0 (0.0)	0 (0.0)	1 (1.9)	0 (0.0)	52
2012.7–9	21 (15.1)	17 (12.2)	19 (13.7)	14 (10.1)	36 (25.9)	5 (3.6)	0 (0.0)	0 (0.0)	0 (0.0)	0 (0.0)	0 (0.0)	3 (2.2)	0 (0.0)	139
2012.10–12	1 (6.7)	1 (6.7)	2 (13.3)	4 (26.7)	1 (6.7)	1 (6.7)	0 (0.0)	0 (0.0)	0 (0.0)	0 (0.0)	0 (0.0)	0 (0.0)	0 (0.0)	15
2013.1–3	0 (0.0)	1 (1.5)	1 (1.5)	4 (6.1)	1 (1.5)	13 (19.7)	0 (0.0)	19 (28.8)	0 (0.0)	0 (0.0)	0 (0.0)	18 (27.3)	0 (0.0)	66
2013.4–6	32 (5.0)	30 (4.7)	38 (5.9)	64 (9.9)	26 (4.0)	286 (44.4)	50 (7.8)	69 (10.7)	0 (0.0)	4 (0.6)	0 (0.0)	51 (7.9)	0 (0.0)	644
2013.7–9	18 (4.5)	17 (4.3)	27 (6.8)	24 (6.0)	24 (6.0)	155 (38.9)	9 (2.3)	49 (12.3)	3 (0.8)	6 (1.5)	0 (0.0)	32 (8.0)	0 (0.0)	398
2013.10–12	15 (5.4)	16 (5.7)	20 (7.1)	15 (5.4)	23 (8.2)	113 (40.4)	10 (3.6)	48 (17.1)	0 (0.0)	2 (0.7)	0 (0.0)	42 (15.0)	0 (0.0)	280
2014.1–3	10 (4.2)	11 (4.7)	13 (5.5)	16 (6.8)	3 (1.3)	112 (47.5)	13 (5.5)	48 (20.3)	1 (0.4)	3 (1.3)	26 (11.0)	19 (8.1)	0 (0.0)	236
2014.4–6	2 (1.7)	2 (1.7)	3 (2.5)	6 (5.0)	5 (4.1)	57 (47.1)	12 (9.9)	19 (15.7)	0 (0.0)	0 (0.0)	8 (6.6)	24 (19.8)	0 (0.0)	121
2014.7–9	16 (5.4)	13 (4.4)	25 (8.4)	10 (3.4)	12 (4.0)	138 (46.3)	10 (3.4)	41 (13.8)	0 (0.0)	1 (0.3)	14 (4.7)	24 (8.1)	0 (0.0)	298
2014.10–12	6 (4.6)	5 (3.8)	5 (3.8)	11 (8.4)	2 (1.5)	63 (48.1)	4 (3.1)	31 (23.7)	2 (1.5)	2 (1.5)	2 (1.5)	28 (21.4)	0 (0.0)	131
2015.1–3	6 (3.1)	7 (3.6)	9 (4.7)	7 (3.6)	2 (1.0)	60 (31.1)	2 (1.0)	57 (29.5)	0 (0.0)	3 (1.6)	1 (0.5)	26 (13.5)	2 (1.0)	193
2015.4–6	7 (2.9)	5 (2.1)	8 (3.3)	16 (6.6)	0 (0.0)	90 (37.3)	19 (7.9)	45 (18.7)	2 (0.8)	0 (0.0)	7 (2.9)	93 (38.6)	0 (0.0)	241
2015.7–9	10 (2.1)	7 (1.4)	16 (3.3)	24 (5.0)	6 (1.2)	171 (35.2)	19 (3.9)	129 (26.5)	0 (0.0)	2 (0.4)	16 (3.3)	161 (33.1)	2 (0.4)	486
2015.10–12	2 (0.5)	16 (3.6)	13 (2.9)	15 (3.4)	1 (0.2)	161 (36.4)	20 (4.5)	151 (34.1)	0 (0.0)	7 (1.6)	3 (0.7)	188 (42.5)	7 (1.6)	442
2016.1–3	7 (3.4)	7 (3.4)	13 (6.3)	7 (3.4)	4 (1.9)	68 (32.9)	8 (3.9)	59 (28.5)	23 (11.1)	3 (1.5)	6 (2.9)	14 (6.8)	0 (0.0)	207
2016.4–6	0 (0.0)	0 (0.0)	2 (1.1)	6 (3.4)	0 (0.0)	62 (34.8)	5 (2.8)	52 (29.2)	18 (10.1)	2 (1.1)	3 (1.7)	35 (19.7)	9 (5.1)	178
2016.7–9	12 (2.1)	18 (3.2)	23 (4.0)	24 (4.2)	2 (0.4)	184 (32.3)	14 (2.5)	126 (22.1)	167 (29.4)	10 (1.8)	10 (1.8)	67 (11.8)	1 (0.2)	569
2016.10–12	7 (2.8)	8 (3.2)	10 (4.0)	10 (4.0)	2 (0.8)	81 (32.7)	10 (4.0)	33 (13.3)	63 (25.4)	2 (0.8)	6 (2.4)	10 (4.0)	0 (0.0)	248
2017.1–3	5 (3.5)	4 (2.8)	6 (4.1)	8 (5.5)	11 (7.6)	45 (31.0)	7 (4.8)	28 (19.3)	39 (26.9)	1 (0.7)	1 (0.7)	13 (9.0)	0 (0.0)	145
2017.4–6	3 (2.5)	7 (5.9)	7 (5.9)	4 (3.4)	3 (2.5)	23 (19.5)	5 (4.2)	29 (24.6)	27 (22.9)	0 (0.0)	2 (1.7)	5 (4.2)	1 (0.9)	118
2017.7–9	0 (0.0)	0 (0.0)	0 (0.0)	0 (0.0)	0 (0.0)	15 (34.9)	2 (4.7)	22 (51.2)	6 (14.)	0 (0.0)	0 (0.0)	4 (9.3)	0 (0.0)	43
Total	424 (5.2)	420 (5.2)	672 (8.3)	1413 (17.5)	294 (3.6)	1999 (24.7)	219 (2.7)	1077 (13.3)	351 (4.3)	49 (0.6)	105 (1.3)	967 (12.0)	22 (0.3)	8091

## Abbreviations

GACVS: Global Advisory Committee of Vaccine Safety
HPV: Human papillomavirus
WHO: World Health Organization
